# Supplementary material for: Comparative multiomics analysis of cell physiological state after culture in a basket bioreactor
Source: Sci Rep. 2022 Nov 23;12:20161. doi: 10.1038/s41598-022-24687-4 (PMC9686226; doi:10.1038/s41598-022-24687-4)
Supplement: Supplementary file 1 — Supplementary Information 1. [file 41598_2022_24687_MOESM1_ESM.zip › raw data/Transcriptomic raw data/7.AS/2.dasplot/TE1vsTC1/A5SS/18_FAM214A_NW_023666048.1_110512987_110513103_-@NW_023666048.1_110513417_110514382_-@NW_023666048.1_110513559_110514382_-.pdf]

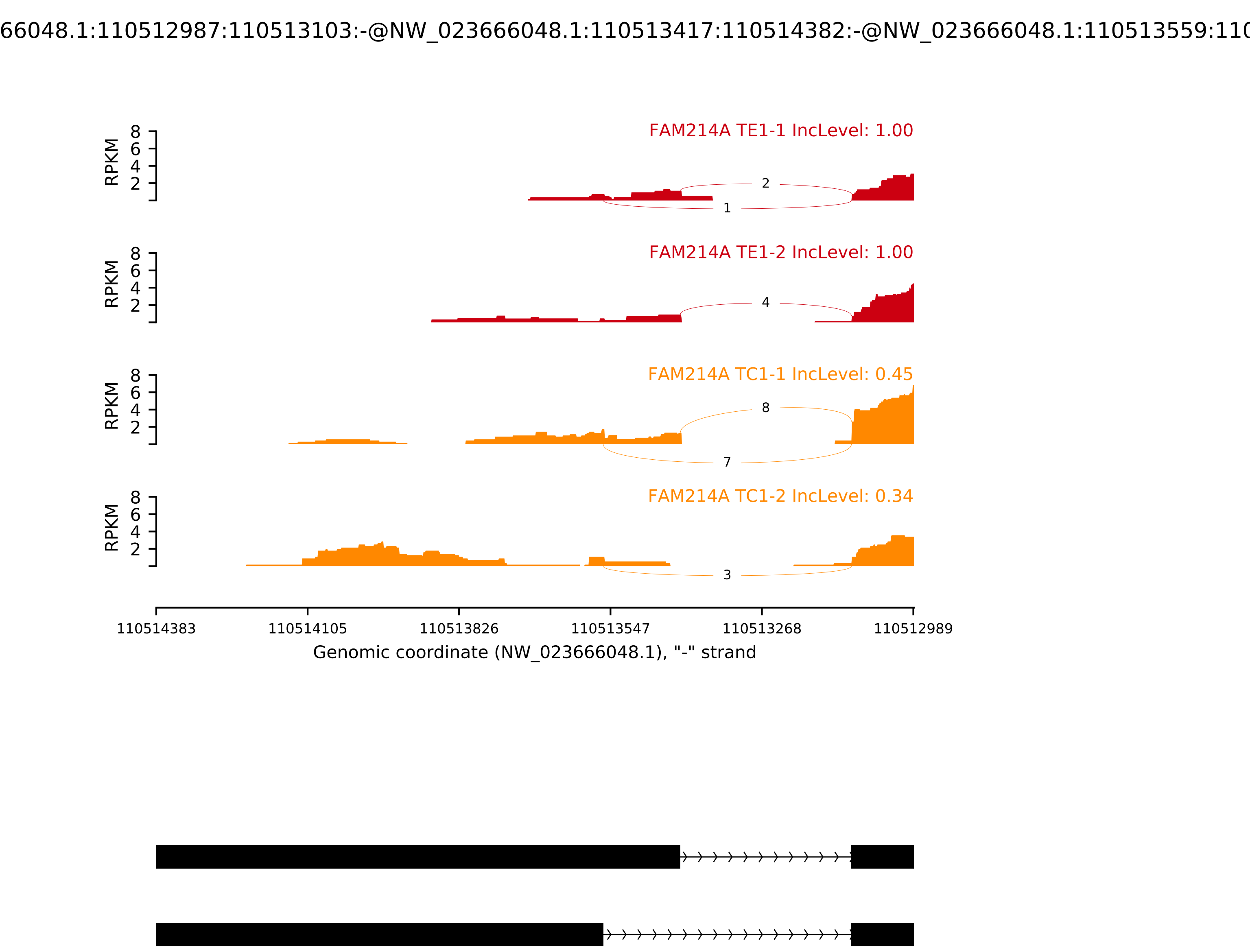
66048.1:110512987:110513103:-@NW_023666048.1:110513417:110514382:-@NW_023666048.1:110513559:110
FAM214A TE1-1 IncLevel: 1.00
FAM214A TE1-2 IncLevel: 1.00
FAM214A TC1-1 IncLevel: 0.45
FAM214A TC1-2 IncLevel: 0.34
RPKM
8
6
4
2
1
2
3
4
7
8
110514383
110514105
110513826
110513547
110513268
110512989
Genomic coordinate (NW_023666048.1), "-" strand
